# Sarcopenia in Endocrine Disorders – The Iceberg or Its Tip?

**DOI:** 10.17925/EE.2015.11.01.41

**Published:** 2015-04-11

**Authors:** Marco A Minetto, Ezio Ghigo

**Affiliations:** 1. Post-doc fellow; 2. Professor of Endocrinology; Director of the School of Medicine, University of Turin, Turin, Italy

**Keywords:** Musculoskeletal system, sarcopenia, tendons

## Abstract

Endocrine myopathies represent disorders of the musculoskeletal system that significantly impair the state of health. Sarcopenia is their pathophysiological common denominator. Recent reports have shown that endocrine disorders, even when subclinical, may also be associated with tendinopathies. It may thus be suggested that both hormones and hormonal disorders have complex actions on the musculoskeletal system and that musculoskeletal endocrinology represents a fascinating research area still awaiting exploration.

Endocrine myopathies represent disorders of the musculoskeletal system that occur in the following pathological conditions: exogenous and endogenous glucocorticoid excess (steroid myopathy), morbid obesity (sarcopenic obesity), hypogonadism, hypothyroidism, hyperparathyroidism, growth hormone deficiency and type 2 diabetes (diabetic myopathy).^1–3^ Because sarcopenia is their pathophysiological common denominator, the musculoskeletal disorders associated with these endocrine diseases have been referred to as endocrine sarcopenic myopathies. They affect at least 10 % of adults in the EU and significantly impair the state of health, especially in elderly subjects, being associated with postural instability, mobility disorders, increased risk of falls and fractures and impaired ability or disability to perform activities of daily living. No widely accepted diagnostic criteria have been established for endocrine sarcopenic myopathies,^2^ so their diagnosis is made either by elimination of other possibilities *(diagnosis per exclusionem)* or by the use of operational criteria developed for the diagnosis of primary sarcopenia. Several consensus groups have recently published operational criteria for the diagnosis of primary sarcopenia (incorporating muscle mass with strength and/or physical performance), including the European Working Group on Sarcopenia in Older People (EGWSOP),^4^ the International Working Group on Sarcopenia (IWGS)^5^ and the Foundation for the National Institutes of Health Sarcopenia Project.^6^ According to EWGSOP criteria,^4^ pre-sarcopenia is defined as the presence of muscle atrophy (reduction of total body or appendicular skeletal muscle mass), sarcopenia is defined as the presence of both muscle atrophy and poor muscle function (low physical performance or low muscle strength) and severe sarcopenia is defined as the presence of muscle atrophy, low physical performance and low muscle strength.

However, the use of operational criteria (and relative cut-points for detection of muscle atrophy) developed for elderly subjects in assessing endocrine patients has not been supported by clinical trials performed in specific patient populations. Moreover, no guidelines have been produced for a disease-specific evaluation and follow-up of endocrine myopathic patients, preventing the objective evaluation of treatments with medicaments, objective testing of new medicaments and accurate quantification of the effects of rehabilitation techniques. In fact, sarcopenia in endocrine myopathies is not a uniform condition, affecting limb muscles more than it does other muscles (e.g., respiratory muscles), lower limb muscles more than upper limb muscles, proximal muscles more than distal muscles and fast muscles more than slow muscles.^7–9^ Accordingly, site-specific assessment of muscle atrophy may be required for early and accurate detection in endocrine patients.

In addition, a unique feature of endocrine myopathies is that they may be associated with tendinopathies, such as rotator cuff disease, patellar tendinopathy and Achilles tendinopathy.^10–13^ The failed healing response represents the pathophysiological mechanism thought to underlie (and not only in endocrine patients) the development of tendinopathies.^14^ However, nothing is known about what really happens in tendons of asymptomatic endocrine patients before they develop a symptomatic tendinopathy. The frequent association between endocrine myopathies and tendinopathies points towards the possible role of hormone disorders (even subclinical) in the pathogenesis of both muscle and tendon disorders. And so a question arises: Is sarcopenic myopathy the predominant side effect of hormonal disorders in the musculoskeletal system? The preponderance of evidence suggests that it is. But if the link with tendinopathies proves valid and reports continue to appear of tendon disorders in association with endocrine and metabolic diseases,^15,16^ sarcopenia may turn out to be only the tip of the iceberg, rendering musculoskeletal endocrinology a fascinating research area still awaiting exploration.
